# Study on pyrolysis gas generation characteristics of insulating oil under electro-thermal action based on ReaxFF simulation

**DOI:** 10.3389/fchem.2026.1866858

**Published:** 2026-07-30

**Authors:** Hua Mao, Jie Wang, Yalong Xia, Zeyao Ma, Fuxiang Li, Xinsheng Lan, Shaoqing Chen, Yan Wang, Xiaoliang Zeng, Qianqian Tian

**Affiliations:** 1 State Grid Sichuan Electric Power Research Institute, Chengdu, Sichuan, China; 2 Power System Security and Operation Key Laboratory of Sichuan, Chengdu, Sichuan, China

**Keywords:** electrical fault, mineral insulating oil, pyrolysis, ReaxFF, thermal fault

## Abstract

Mineral insulating oil serves as the core insulation and cooling medium of power transformers, and its pyrolysis and gas generation behavior directly determine the safe operation of equipment. Conventional dissolved gas analysis (DGA) only targets small-molecule C1/C2 gases produced in the late pyrolysis stage, which inevitably causes obvious delay in early fault identification. In this work, ReaxFF reactive force field molecular dynamics simulations and physical experiments are jointly adopted to build a multi-component insulating oil molecular model consisting of alkanes, cycloalkanes and aromatics. We systematically investigate oil pyrolysis under single thermal stress and coupled electro-thermal stress, with C_3_ and C_4_ hydrocarbon intermediates as the exclusive core monitoring indicators. Simulation results reveal that higher temperature accelerates overall oil pyrolysis; C1 and C2 dominate final pyrolysis products, while C_3_ accumulates massively at moderate temperatures and C4 appears in trace quantities specifically in the medium-temperature window. The superimposed electric field supplies extra reaction energy and facilitates the generation of C3, C4 as well as small-molecule gases. Consistent experimental observations further characterize the unique evolutionary patterns of C3/C4: under thermal faults, C3 and C4 intermediate concentrations follow a distinct rise-then-fall trend with growing temperature; under discharge faults, total gas yield rises with applied voltage, yet C4 content declines synchronously. Notably, significant concentration fluctuations of C3 and C4 can be captured under non-breakdown incipient fault states, long before prominent changes of conventional C1/C2 gases. This study fully demonstrates that continuous monitoring of the characteristic variation rules of C3 and C4 intermediates can effectively reflect the early pyrolysis signatures of insulating oil, offering a novel and reliable basis for early warning of latent transformer faults.

## 1 Introduction

As a critical insulation, cooling and arc-extinguishing medium in power transformers, the thermal and chemical stability of insulating oil is directly related to the safe operation and service life of power equipment (Wang et al., 2002). In actual operation, internal faults such as local overheating and discharge in transformers often cause pyrolysis of insulating oil under electro-thermal coupling, generating small-molecule characteristic gases including hydrogen and methane (Raj et al., 2024). These pyrolysis gases gradually dissolve in the insulating oil during transformer operation (Jiang et al., 2024). With the development of faults and prolonged operation, gases accumulate continuously in the oil, inducing local overheating and even catastrophic accidents such as electrical breakdown or equipment explosion in severe cases (Thiviyanathan et al., 2022). Traditional fault diagnosis for insulating oil mainly depends on DGA, which judges the fault type and severity by detecting the content changes of final products such as methane and acetylene (Mao et al., 2025). However, these final products usually appear in the late stage of pyrolysis, and their generation and accumulation require certain reaction time and energy input, resulting in a time lag in diagnostic results and making it difficult to achieve early fault warning (Cai et al., 2026). Therefore, exploring intermediate products that can reflect the initial stage of pyrolysis earlier is of great significance for improving the timeliness and accuracy of fault diagnosis.

With the development of computer technology, molecular dynamics simulation based on the ReaxFF reactive force field has gradually become an important method to study the pyrolysis mechanism of insulating oil (Van Duin et al., 2001). This method can simulate the breaking and formation of chemical bonds at the atomic level and reveal the evolution laws of various products during pyrolysis (Yang et al., 2024; Zhou and Huang, 2010). Li et al. (2025) used molecular dynamics simulation to compare and analyze the decomposition of gas-to-liquid (GTL) oil and mineral oil under electro-thermal coupling faults, finding that the proportion of H_2_ and CH_4_ produced by GTL oil decomposition is about 5% higher than that of mineral oil. Huang et al. (2021) simulated the high-temperature pyrolysis of mineral insulating oil at 2,200 K–2,800 K, revealing the sequential pyrolysis law of alkanes, cycloalkanes and aromatics. Wang Wujing et al. (Du et al., 2018). Adopted reactive molecular dynamics simulation to reveal the main reaction paths of mineral insulating oil pyrolysis and the formation mechanism of small-molecule characteristic gases. Jakob et al. (2011) proposed an energy-weighted DGA method. In transformer oil, electrical faults mainly include partial discharge, spark discharge and arc discharge, with increasing fault severity. At present, research on electrical faults is mainly carried out based on three typical electrode discharge experiments and molecular dynamics simulations.

At present, research on the pyrolysis and gas production behavior of mineral insulating oil under electro-thermal coupling is insufficient, especially the attention paid to medium and high-carbon hydrocarbon components generated during pyrolysis is relatively limited. Based on the ReaxFF-MD method, this study constructs a molecular model of typical mineral insulating oil, simulates its pyrolysis process under thermal stress and electro-thermal coupling, focuses on the formation paths and evolution laws of intermediates such as C3 and C4, and analyzes their correlation mechanism with the early stage of pyrolysis. Through comparative study with final products, the feasibility of using intermediates as early characteristic gases for faults is discussed, providing a theoretical basis for real-time monitoring and early warning of the insulation state of transformers.

## 2 Insulating oil pyrolysis theory and ReaxFF principle

### 2.1 Insulating oil pyrolysis theory

Mineral insulating oil is mainly composed of three types of hydrocarbons: alkanes (C_n_H_2n+2_, mass fraction ∼60%), cycloalkanes (C_n_H2n, mass fraction 10%–40%) and aromatics (C_n_H_2n-6_, mass fraction 5%–15%). In addition, it contains a small amount of high-molecular compounds and organic substances such as organic acids, sulfides and asphaltenes. The average carbon number of its molecules is 19–23, and the average molecular weight is about 270–310 g/mol (Rouse, 2002). The pyrolysis process of mineral insulating oil mainly involves complex chemical reactions such as chain scission, dehydrogenation, ring opening and condensation of various hydrocarbons. When thermal or electrical faults occur in oil-immersed transformers, a large amount of energy is released in local areas, causing a rapid temperature rise at fault points. This breaks the C—H and C—C bonds in long-chain molecules of nearby mineral insulating oil, generating a small number of hydrogen free radicals (H^+^) and small-molecule hydrocarbon free radicals (e.g., CH_3_·, CH_2_, CH·) (Wang et al., 2018). These high-energy hydrogen free radicals and hydrocarbon free radicals react with each other, finally producing characteristic gases such as hydrogen (H_2_), methane (CH_4_), acetylene (C_2_H_2_), ethylene (C_2_H_4_) and ethane (C_2_H_6_) (Mariprasath and Ravindran, 2022).

### 2.2 ReaxFF principle

Proposed by Van Duin et al. (2001), the ReaxFF reactive force field can effectively describe the breaking and formation of chemical bonds in hydrocarbon compounds, providing a reliable and efficient calculation method for large-scale molecular dynamics simulations of complex reaction systems. The potential energy curve calculated by the ReaxFF reactive force field is in good agreement with the results of density functional theory (DFT), and the calculation time is reduced by about one order of magnitude. Compared with quantum mechanical methods, ReaxFF molecular simulation can spontaneously determine reaction paths driven by the evolution of the system without preset reaction coordinates, making it more suitable for the study of complex reaction systems (Wang et al., 2019).

In the ReaxFF force field, the potential energy function formula is:

Esystem=Ebond+Eover+Eunder+Eval+Epen+Etor+Econj+EvdWaals+ECoulomb

The potential energy function consists of four main parts: bond energy Ebond; bond angle energies Eval and Epen; torsion energy Etor and conjugation energy Econj; non-bonding forces EvdWaals and ECoulomb. In addition, there are correction terms Eover and Eunder for energy correction to reduce calculation errors. The ReaxFF reactive force field calculates bond orders based on interatomic potential energy and judges the formation and breaking of chemical bonds by tracking real-time changes in bond orders, thus simulating complex chemical reactions without preset reaction paths.

## 3 ReaxFF simulation and electro-thermal experiments

### 3.1 Simulation

**FIGURE 1:**
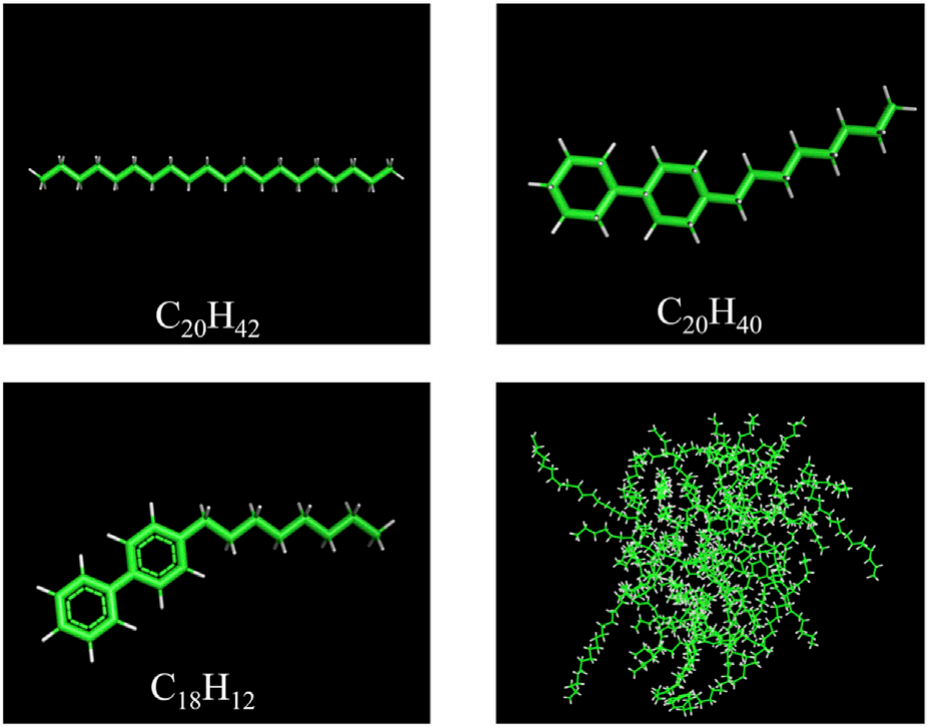
Geometric model of the simulated molecule.

The initial structure for each reaction system was created by randomly placing 24 alkanes (C_20_H_42_), 12 cycloalkanes (C_20_H_40_) and 4 aromatic hydrocarbons (C_18_H_12_) inside a 45.3 Å cubic box using the Packmol program (Martínez et al., 2009), ensuring a minimum spacing of 2.0 Å between any two molecules (As shown in Figure 1). The thermal decomposition of the hydrocarbon mixture was then carried out using the reactive force field (ReaxFF) as implemented in LAMMPS (Thompson et al., 2022), employing thr parameters with C and H entries from Castro-Marcano et al. (2012), Weismiller et al. (2010), and Chenoweth et al. (2008). Before simulation runs, the energy was minimized until both the total energy change and the maximum force were below 1 × 10^−10^ kcal/mol and 1 × 10^−10^ kcal/mol/Å, respectively. These ReaxFF simulations used the isothermal-isochoric (NVT) ensemble with non-periodic condition at x direction and periodic boundary conditions at y/z directions, and were performed for 10,000,000 steps (1 ns). Nine thermodynamic states were studied: 2,500, 3,000, 3,500 and 4,000 K without electric field, and 3,000 K with electric fields of 1, 2, 3, 4 and 5 MV/m applied along the x-axis. The field was implemented as a uniform force acting on each atom with charge. During all simulations, the time step was fixed at 0.10 fs. The bond order cut-off was set at 0.30, meaning two atoms were considered unbonded if their bond order fell below this value; this same threshold was used to identify chemical bonds when counting molecules.

### 3.2 Insulating oil pyrolysis analysis experiment

In the thermal analysis experiment of insulating oil, Karamay No.25 transformer oil from Xinjiang was selected as the research object. The sample was dried and not aged to ensure its original physical and chemical properties. A resistance heating method was used to gradually heat 1 L of insulating oil sample to 100 °C–400 °C under controlled conditions, simulating different degrees of thermal faults that may occur in transformers during actual operation, and investigating its thermal stability and pyrolysis behavior.

**FIGURE 2:**
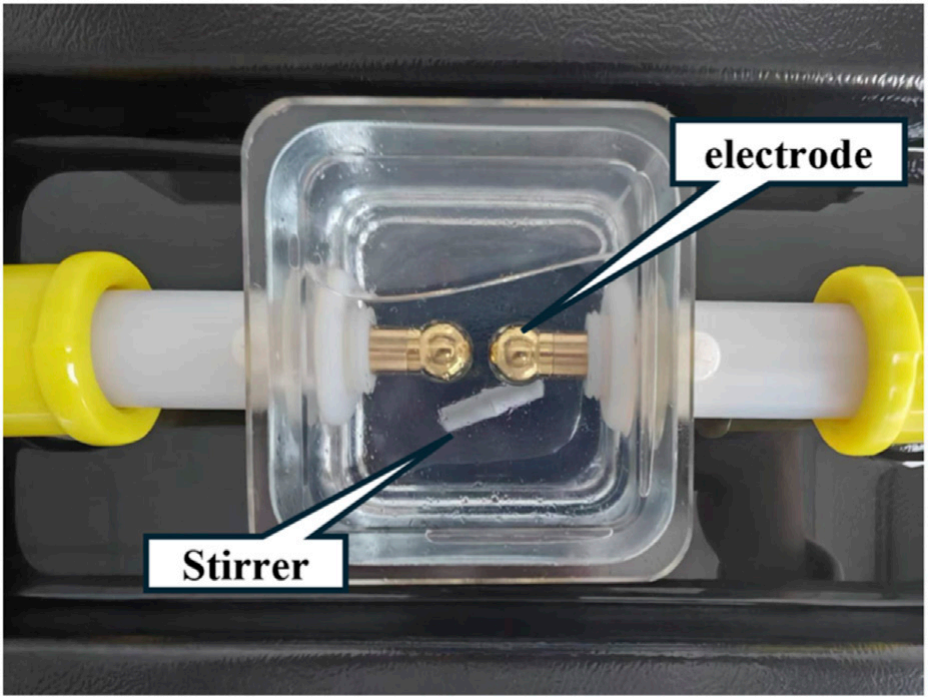
Electrical fault simulation device.

In addition, to explore the evolution characteristics of insulating oil under electrical fault conditions, a custom electrical fault simulation cell as shown in Figure 2 was adopted. The electrodes are spherical with a diameter ranging from 12.5 mm to 13.0 mm, and the electrode gap is set to 2.5 mm ± 0.5 mm. A volume of 150 mL insulating oil with uniform specifications was poured into the oil cup for each test. Voltage loading experiments were conducted at partial discharge magnitudes of 600 pC, 1,000 pC, 1,500 pC and 2,000 pC. By applying graded voltage stress, we analyzed the decomposition features and gas generation laws of insulating oil under electric field excitation, which provides solid experimental support for revealing the formation mechanism of dissolved gases in transformer oil.

## 4 Analysis of experimental results

### 4.1 Analysis of thermal fault cracking in insulating oil

#### 4.1.1 Thermal pyrolysis simulation experiment

**FIGURE 3:**
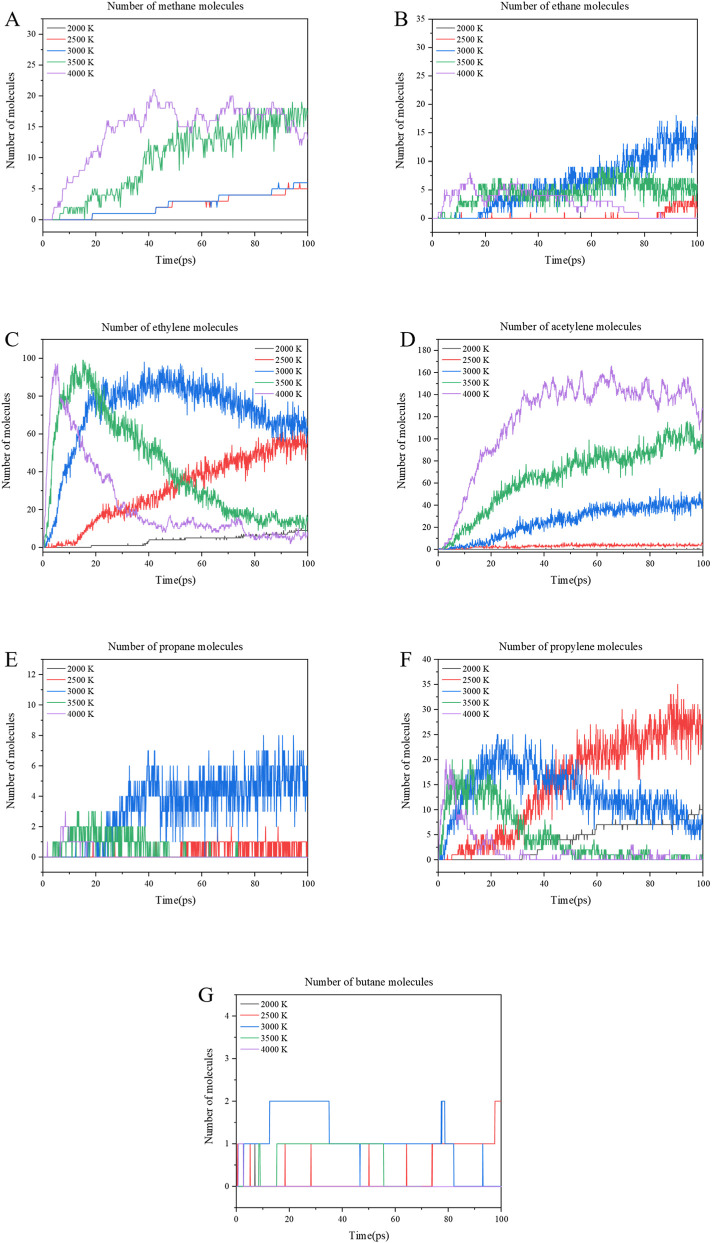
Number of gas molecules produced by thermal decomposition of insulating oil **(A)** Number of methane molecules **(B)** Number of ethane molecules **(C)** Number of ethylene molecules **(D)** Number of acetylene molecules **(E)** Number of propane molecules **(F)** Number of propylene molecules **(G)** Number of butane molecules.

Based on previous research conclusions (Wang et al., 2021), reaction temperature mainly affects the chemical reaction rate without changing the reaction path or mechanism. Generally, the increase of temperature helps to overcome the reaction energy barrier, thus significantly accelerating the reaction process. To effectively observe the pyrolysis behavior of insulating oil components and obtain statistically significant simulation results with limited computing resources, this study appropriately increased the simulation temperature to shorten the characteristic reaction time on the premise of ensuring no change in reaction mechanism (Castro-Marcano et al., 2012). Therefore, the simulation temperatures under the ReaxFF force field were set to 2,000 K, 2,500 K, 3,000 K, 3,500 K and 4,000 K respectively, and a series of comparative simulation experiments were carried out to systematically investigate the influence of temperature on the evolution law of pyrolysis products of insulating oil. The results are shown in Figure 3.

This study simulated the time-dependent variations in the molecular counts of pyrolysis products, including methane, ethane, ethylene, acetylene, propane, propylene, and butane, from insulating oil at temperatures ranging from 2,000 to 4,000 K. At the low-temperature stage (2,000 K), the pyrolysis extent was weak, and the molecular counts of all products were nearly zero. As the temperature increased to 2,500–4,000 K, the pyrolysis reaction was significantly enhanced. Specifically, the molecular count of methane increased markedly at temperatures of 3,500 K and above; ethylene reached its peak yield at 3,000 K; and acetylene rose sharply from 2,500 K and achieved the maximum yield at 4,000 K. C3 products (propane and propylene) were steadily generated at 3,000 K, but exhibited a distinct decreasing trend in the later reaction stage with rising temperature and prolonged reaction time.

These results indicate that at relatively low energy levels, insulating oil pyrolysis mainly produces methane and ethylene with high yields. As the temperature and input energy increase, the yield of acetylene rises gradually. C3 and C4 products mainly exist in the medium-low temperature range and show an obvious trend of increasing first and then decreasing with rising temperature.

#### 4.1.2 Insulating oil thermal fault test

**FIGURE 4:**
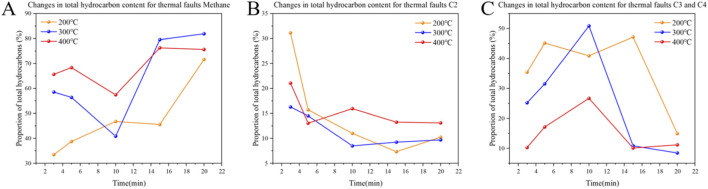
Changes in gas content of different thermal faults at various temperatures **(A)** Changes in total hydrocarbon content for thermal faults Methane **(B)** Changes in total hydrocarbon content for thermal faults C2 **(C)** Changes in total hydrocarbon content for thermal faults C3 and C4.

To verify the reliability of the simulation results, systematic thermal fault experiments were performed on mineral insulating oil. Aiming to reproduce the local overheating faults encountered by transformers in field service, the oil samples were heated to predetermined temperatures and maintained for various holding times to examine the influence of thermal effects on oil pyrolysis. Following the heating process, the composition of generated gas products was analyzed, and the evolution characteristics of pyrolysis products under different thermal stresses were systematically investigated. The results are shown in Figure 4.

As shown in the figures, the proportion of methane increases with rising temperature and prolonged reaction time and is significantly higher in the 300 °C and 400 °C groups than in the 200 °C group. C2 species account for a high proportion initially but decrease over time and eventually stabilize. The proportions of C3 and C4 reach up to 50% in the middle stage of the fault and decline to approximately 10% with increasing time. It can be inferred that during the pyrolysis of insulating oil, a large amount of C1 and C2 species are mainly generated at low temperatures. With the elevation of temperature and energy input, the relative content of C3 and C4 intermediates increase gradually. As the energy rises further, C3 and C4 species become unstable and decompose into C1 and C2 hydrocarbons.

Simulation and experimental results demonstrate that at moderate and low temperatures, the yields of methane and ethylene are relatively high. As the temperature increases, the energy acquired by insulating oil rises, and the acetylene content increases gradually, eventually leading to a significant rise in its proportion. C3 and C4 gases are generated in trace amounts at the low-temperature stage, then increase progressively with elevated temperature and prolonged duration. When the temperature rises further, C3 and C4 gases show a decreasing trend. The experimental results are generally consistent with simulation results, and these intermediates can provide effective support for insulating oil fault diagnosis.

#### 4.1.3 Inference of the thermal fault cracking mechanism of insulating oil

**FIGURE 5:**
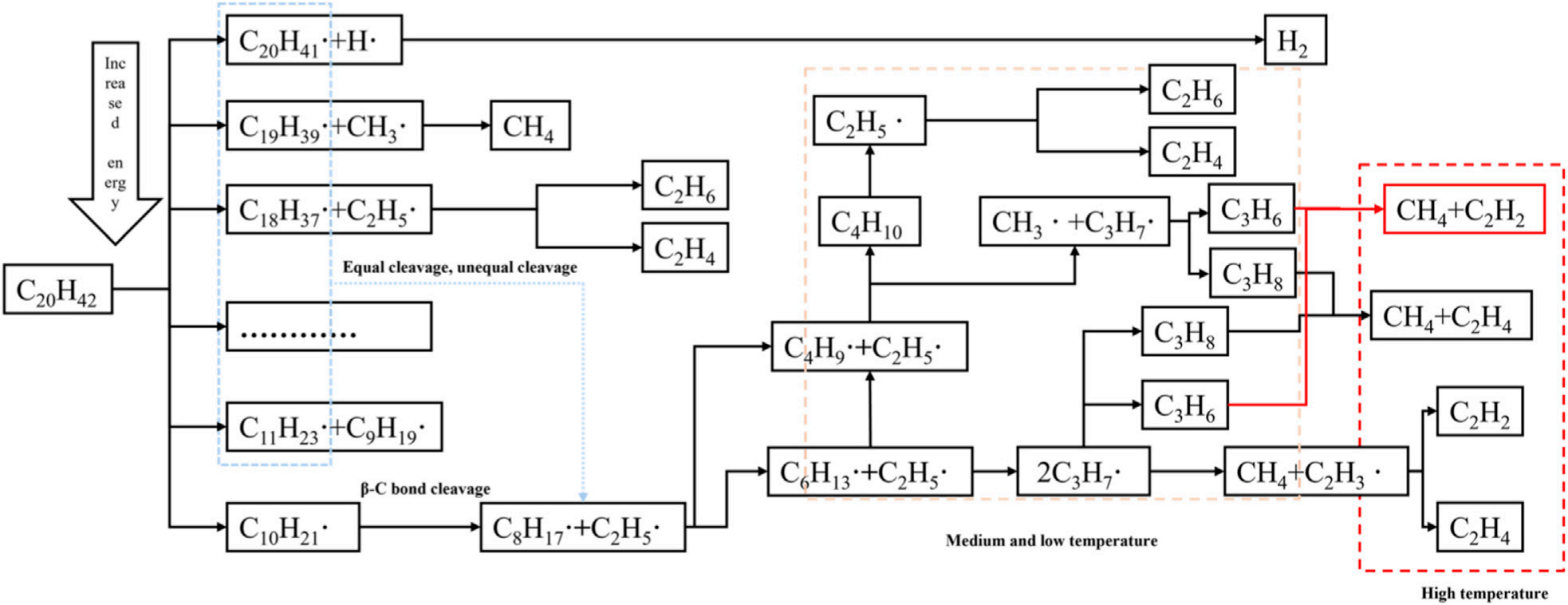
Thermal fault cracking pathways of insulating oil.

Based on the analytical results, n-alkane C_20_H_42_ was selected as a representative example in this study. In the simulation, carbon and hydrogen atoms were distinguished by different colors; by observing the dynamic visualization of the mineral oil pyrolysis process and combining the findings with experimental results, the reaction pathways and formation processes of all products were deduced. The primary reaction pathways are illustrated in Figure 5 below.

In the initial stage, homolytic and heterolytic cleavage of C-C or C-H bonds occurs in long-chain alkane molecules, preferentially via β-carbon scission to generate alkyl radicals of varying chain lengths (e.g., C_10_H_21_·, C_8_H_17_·) and small molecular radicals (H·, CH_3_·, C_2_H_5_·). These primary radicals rapidly undergo disproportionation and combination reactions, directly producing initial characteristic gaseous products such as C_3_H_7_· and C_2_H_5_·. In the moderate-temperature range, medium-chain alkyl radicals (e.g., C_6_H_13_·, C_4_H_9_·) further fragment into short-chain species (C_3_H_7_, C_2_H_5_·), which participate in secondary reactions to form light hydrocarbons including C_3_H_8_, C_3_H_6_, and C_2_H_4_. The products are dominated by saturated and low-unsaturated hydrocarbons, corresponding to early-stage faults such as low-temperature overheating or partial discharge in insulating oil. At high temperatures, light hydrocarbons undergo extensive dehydrogenation and acetylenation reactions, with the vinyl radical (C_2_H_3_·) acting as a key intermediate, ultimately leading to the formation of highly unsaturated acetylene (C_2_H_2_), which signifies the occurrence of high-energy faults such as arc discharge.

### 4.2 Insulating oil electrical fault decomposition analysis

#### 4.2.1 Electro-thermal coupling pyrolysis experiment

**FIGURE 6:**
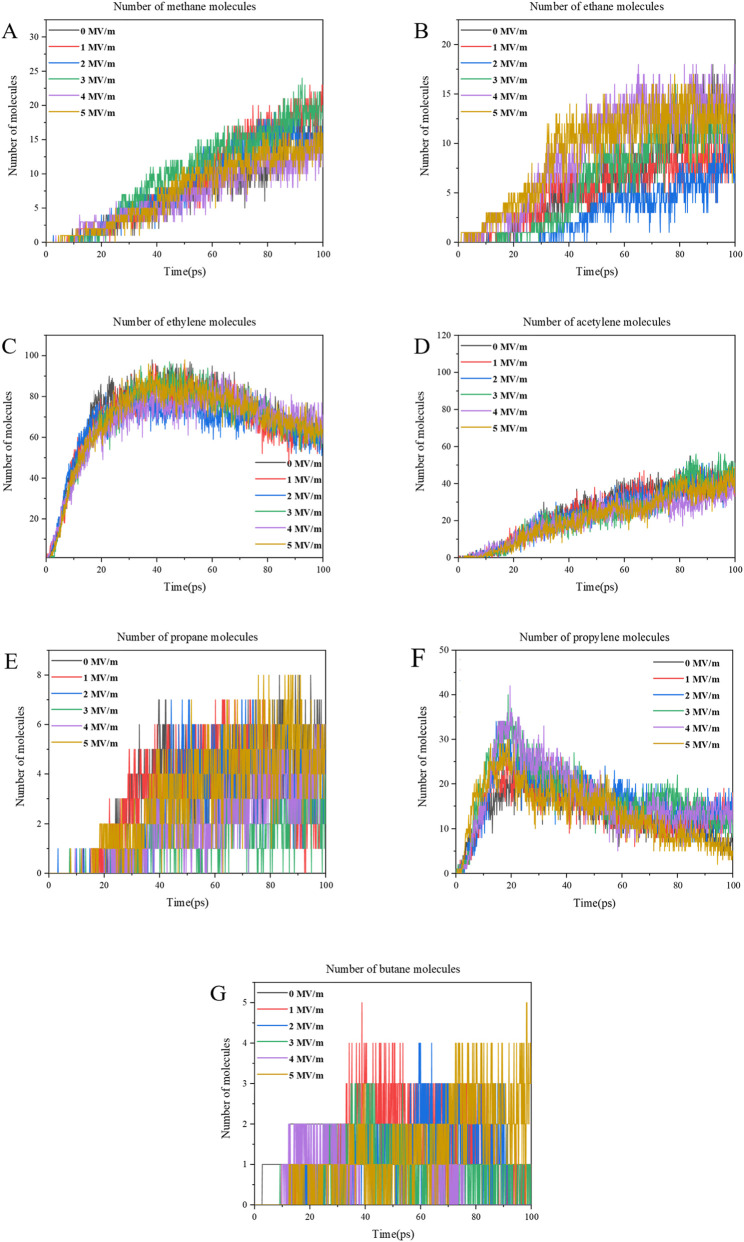
Number of gas molecules in insulating oil electrical decomposition **(A)** Number of methane molecules **(B)** Number of ethane molecules **(C)** Number of ethylene molecules **(D)** Number of acetylene molecules **(E)** Number of propane molecules **(F)** Number of propylene molecules **(G)** Number of butane molecules.

In practical operation, besides thermal faults, electrical discharge is also a crucial factor triggering insulating oil pyrolysis and is even more common. For this reason, in this study, simulations were performed at a fixed temperature of 3,000 K with electric field intensities of 1, 2, 3, 4, and 5 MV/m applied separately, to systematically investigate the influence of electric field strength on the pyrolysis behavior of insulating oil. The results are shown in Figure 6.

According to the ReaxFF molecular dynamics simulation results, the molecular counts of gas products generated from electro-thermal pyrolysis of insulating oil under different electric field intensities all exhibit a trend of rapid increase followed by gradual stabilization over time. The yields of small-molecule C1–C2 hydrocarbons increase significantly with increasing electric field intensity, among which ethylene and acetylene are highly sensitive to the electric field and become the dominant products under high electric fields. Propane is less affected by the electric field, while the yield of propylene increases with elevated electric field intensity, and butane exists only in small amounts under low electric fields.

The results illustrate that the electric field provides additional energy and promotes the formation of primary C1 and C2 products. As the electric field intensity increases, the initiation time of C–C bond cleavage in long-chain hydrocarbons is advanced and the reaction rate is accelerated, leading to a corresponding increase in the peak molecular counts of final products. The electric field exerts an indirect promotional effect on C3 intermediates: a high electric field intensity enhances the formation of intermediates, which serve as sufficient precursors for the generation of C1 and C2 species, thereby elevating their concentrations while accelerating both the production and subsequent decomposition of C3 intermediates. For by-products such as butane (C4 species), the electric field also shows a certain promotional effect; however, due to the high tendency of C4 species to decompose, their concentrations remain at a low level throughout the reaction. Overall, a higher electric field intensity promotes product formation, accelerates the overall pyrolysis process, and simultaneously increases the total yield of all pyrolysis products.

#### 4.2.2 Insulating oil electrical fault experiment

**FIGURE 7:**
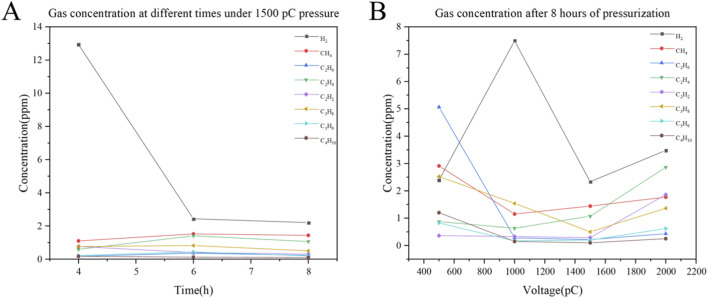
Variation of Gas Concentrations in Electrical Faults **(A)** Gas concentration at different times under 1,500 pC pressure **(B)** Gas concentration after 8 h of pressurization.

To verify the reliability of the simulation results, electrical fault experiments on insulating oil were further conducted. Aiming to reproduce the gas generation process of transformer oil under actual operating conditions, a fully enclosed fault simulation device was employed in this study to establish a controllable electrical stress environment without causing breakdown of the oil samples, so as to restore the real pyrolysis behavior of insulating oil under fault conditions. During the experiments, characteristic gases generated in the oil samples were collected and measured, and their composition and content variations were systematically analyzed to reveal the pyrolysis mechanism of insulating oil under different fault modes. The results are shown in Figure 7.

When the discharge magnitude was below 1,500 pC, the concentrations of methane, ethane, ethylene, and acetylene exhibited a trend of initial decrease followed by increase, whereas propane, propylene, and butane declined continuously. When the discharge magnitude exceeded 1,500 pC without oil breakdown, the contents of all gas products began to rise. These results indicate that under moderate-to-high discharge levels, C3 and C4 products undergo progressive bond-cleavage reactions with increasing discharge intensity and decompose into C1 and C2 gases. In the high discharge range, the chain-scission reactions of insulating oil become highly active, resulting in massive gas generation.

Both simulation and experimental results demonstrate that the ReaxFF simulation outcomes are in good agreement with the electro-pyrolysis experimental results of insulating oil. Both indicate that an increase in external field intensity can significantly accelerate the pyrolysis of insulating oil and promote bond cleavage and deep pyrolysis of long-chain hydrocarbons. With increasing discharge magnitude or electric field strength, the yields of small-molecule C1 and C2 hydrocarbons increase notably, and unsaturated hydrocarbons such as ethylene and acetylene are particularly sensitive to the electric field environment. C3 and C4 intermediates maintain relatively high contents under low field conditions, but their concentrations decrease under high fields due to continuous decomposition, showing an overall trend of conversion into small-molecule hydrocarbons. The introduction of the electric field accelerates the pyrolysis process; chain scission reactions proceed more rapidly under high electric fields, leading to a pronounced increase in total gas production. These combined results confirm that C3 and C4 intermediates can be used as auxiliary characteristic gases for fault diagnosis of insulating oil.

#### 4.2.3 Inference of the mechanism of electrical fault cracking in insulating oil

**FIGURE 8:**
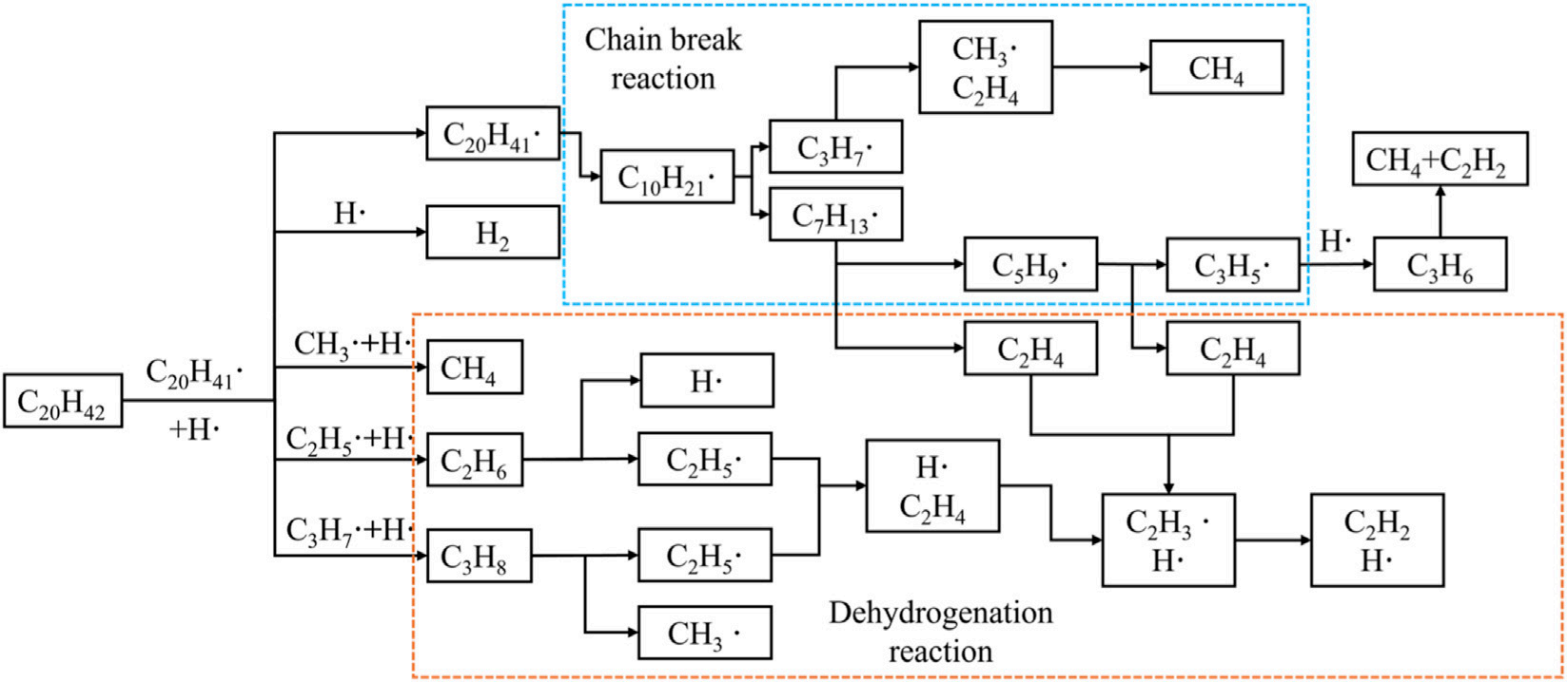
Insulating oil electrical fault decomposition pathway.

On the basis of the above analysis, n-alkane C_20_H_42_ was employed as a model compound, and carbon and hydrogen atoms were marked with different colors in the simulations. By visualizing the dynamic evolution of mineral oil pyrolysis and combining with experimental data, the reaction pathways and formation mechanisms of various products were deduced. The main reaction routes are illustrated in Figure 8.

The electro-pyrolysis of insulating oil is initiated mainly by dehydrogenation. Under the action of a high-energy electric field, molecules undergo dehydrogenation: hydrogen atoms detach from the molecular chains and exist as free radicals, while the parent molecules are converted into long-chain alkyl radicals such as C_20_H_41_·, accompanied by the formation of small-molecule products including H_2_, CH_4_, C_2_H_6_, and C_3_H_8_. Subsequently, long-chain alkyl radicals such as C_20_H_41_· undergo continuous chain scission via β-cleavage, gradually transforming into medium- and short-chain radicals including C_10_H_21_·, C_3_H_7_·, and C_7_H_13_·. During this process, dehydrogenation and chain scission occur repeatedly, leading to the continuous generation and conversion of intermediates. C_3_-related radicals such as C_3_H_7_·, C_5_H_9_·, and C_3_H_5_· further produce C_3_-type characteristic gases (e.g., C_3_H_6_) through dehydrogenation and recombination reactions. Meanwhile, short-chain radicals such as C_2_H_5_· and C_2_H_3_· are successively converted into unsaturated hydrocarbons including C_2_H_4_ and C_2_H_2_ via continuous dehydrogenation. The combination of CH_3_· and H· finally yields CH_4_.

Overall, dehydrogenation persists throughout the entire electro-pyrolysis process of insulating oil. The proposed mechanism reveals the evolutionary pathway from C_20_H_42_ to characteristic gases such as CH_4_, C_2_H_4_, C_2_H_2_, and C_3_H_6_ through sequential dehydrogenation and chain scission, and elucidates the microscopic reaction mechanism of electric-field-driven electro-pyrolysis of insulating oil at the molecular level.

## 5 Conclusion

This work carries out a combined experimental and ReaxFF molecular dynamics simulation study on insulating oil pyrolysis under electro-thermal coupling stress, with special emphasis on the characteristic evolution laws of C3 and C4 intermediate hydrocarbons. The results demonstrate distinct regular variation patterns of C3 and C4 under different fault types: Under electrical faults, the growth of electric field intensity and discharge magnitude drastically accelerates the bond scission and deep pyrolysis of long-chain alkane molecules, which continuously consumes the generated C3 and C4 intermediates and converts them into C1 and C2 small-molecule gases. Although unsaturated gases like ethylene and acetylene show strong responsiveness to high electric field energy and total gas output rises sharply with enhanced field stress, C3 and C4 contents continuously decline as discharge intensity intensifies. Under single thermal fault conditions, temperature rise also promotes oil pyrolysis. Methane and ethylene dominate low-temperature products, while acetylene occupies a larger share at high temperatures. Most notably, C3 and C4 intermediates follow an obvious and universal concentration trend of increasing first and then decreasing as temperature climbs. This unique rise-then-fall profile is the intrinsic marker proving that C3 and C4 act as transient intermediate products during the pyrolysis cascade reaction.

Consistent trends are observed between experimental and simulation results under both thermal and electrical faults, which validates that C3 and C4 hydrocarbons serve as important intermediates during insulating oil pyrolysis. These species can be incorporated into the diagnostic system as auxiliary characteristic gases to improve the accuracy and reliability of transformer fault diagnosis, providing a relatively comprehensive theoretical basis for the insulation condition assessment of power equipment.

## Data Availability

The raw data supporting the conclusions of this article will be made available by the authors, without undue reservation.

## References

[B1] Chenoweth K. Van Duin A. C. T. Goddard W. A. (2008). ReaxFF reactive force field for molecular dynamics simulations of hydrocarbon oxidation. J. Phys. Chem. A 112 (5), 1040–1053. 10.1021/jp709896w 18197648

[B2] Cai C. Zhou B. Wang X. Chen C. Feng S. Cheng P. (2026). Numerical simulation of two-phase gas generation and propagation in transformer oil under arc faults. IEEE Access 14, 42285–42293. 10.1109/access.2026.3671308

[B3] Castro-Marcano F. Kamat A. M. Russo M. F. Jr van Duin A. C. Mathews J. P. (2012). Combustion of an Illinois no. 6 coal char simulated using an atomistic char representation and the ReaxFF reactive force field. Combust. Flame 159 (3), 1272–1285. 10.1016/j.combustflame.2011.10.022

[B4] Du L. Wang W. J. Zhang B. D. Wang Y. B. Xiang C. M. Wang J. (2018). Molecular dynamics simulation of mineral insulating oil pyrolysis based on ReaxFF. High. Volt. Eng. 44 (2), 488–497. 10.13336/j.1003-6520.hve.20180131020

[B5] Huang H. Wang X. Z. Deng X. P. Liu B. (2021). “Molecular dynamics simulation on high temperature pyrolysis of transformer insulating oil under thermal stress,” in IEEE 5th Conference on Energy Internet and Energy System Integration, 3730–3734. 10.1109/EI252483.2021.9713582

[B6] Jakob F. Noble P. Dukarm J. J. (2011). A thermodynamic approach to evaluation of the severity of transformer faults. IEEE Trans. Power Deliv. 27 (2), 554–559. 10.1109/tpwrd.2011.2175950

[B7] Jiang Z. Li X. Zhang H. Zhang E. Liu C. Fan X. (2024). Research progress and prospect of condition assessment techniques for oil–paper insulation used in power systems: a review. Energies 17 (9), 2089. 10.3390/en17092089

[B8] Li X. Chen B. Ma Y. Liu J. Yang M. (2025). Differential analysis of gas generation between gas to liquid and mineral oil under electric-thermal combined fault based on molecular dynamics. Electr. Eng. 107 (6), 7457–7465. 10.1007/s00202-024-02846-2

[B9] Mao H. Wang F. Wang J. Ma Z. Wang Y. Peng R. (2025). Research progress of online monitoring techniques for dissolved gas analysis in insulating oil. Front. Chem. 13, 1721893. 10.3389/fchem.2025.1721893 41479587 PMC12753880

[B10] Mariprasath T. Ravindran M. (2022). An experimental study of partial discharge analysis on environmental friendly insulating oil as alternate insulating material for transformer. Sādhanā 47 (4), 204. 10.1007/s12046-022-01946-8

[B11] Martínez L. Andrade R. Birgin E. G. Martínez J. M. (2009). PACKMOL: a package for building initial configurations for molecular dynamics simulations. J. Comput. Chem. 30 (13), 2157–2164. 10.1002/jcc.21224 19229944

[B12] Raj R. A. Sarathkumar D. Sidhik A. S. Annamalai A. Venkatachary S. K. Andrews L. J. B. (2024). A short review on the key indicators of ageing studies conducted on insulating oil. IEEE international students' conference on electrical, Electronics and Computer Science, 1–6. 10.1109/SCEECS61402.2024.10482003

[B13] Rouse T. O. (2002). Mineral insulating oil in transformers. IEEE Electr. Insul. Mag. 14 (3), 6–16. 10.1109/57.675572

[B14] Thiviyanathan V. A. Ker P. J. Leong Y. S. Abdullah F. Ismail A. Zaini Jamaludin M. (2022). Power transformer insulation system: a review on the reactions, fault detection, challenges and future prospects. Alexandria Eng. J. 61 (10), 7697–7713. 10.1016/j.aej.2022.01.026

[B15] Thompson A. P. Aktulga H. M. Berger R. Bolintineanu D. S. Brown W. M. Crozier P. S. (2022). LAMMPS-a flexible simulation tool for particle-based materials modeling at the atomic, meso, and continuum scales. Comput. Physics Communications 271, 108171. 10.1016/j.cpc.2021.108171

[B16] Van Duin A. C. T. Dasgupta S. Lorant F. Goddard W. A. (2001). ReaxFF: a reactive force field for hydrocarbons. J. Phys. Chem. A 105 (41), 9396–9409. 10.1021/jp004368u

[B17] Wang X. Tang C. Huang B. Hao J. Chen G. (2018). Review of research progress on the electrical properties and modification of mineral insulating oils used in power transformers. Energies 11 (3), 487. 10.3390/en11030487

[B18] Wang M. Vandermaar A. J. Srivastava K. D. (2002). Review of condition assessment of power transformers in service. IEEE Electr. Insul. Mag. 18 (2), 12–25. 10.1109/mei.2002.1161455

[B19] Wang X. Xu W. Zhu W. Li Q. m. (2019). Reactive molecular dynamics simulation of transformer oil pyrolysis based on ReaxFF reactive force field. IOP Conf. Ser. Mater. Sci. Eng. 486 (1), 012029. 10.1088/1757-899x/486/1/012029

[B20] Wang Y. Gong S. Wang H. Li L. Liu G. (2021). High-temperature pyrolysis of isoprenoid hydrocarbon p-menthane using ReaxFF molecular dynamics simulation. J. Anal. Appl. Pyrolysis 155, 105045. 10.1016/j.jaap.2021.105045

[B21] Weismiller M. R. Van Duin A. C. T. Lee J. Yetter R. A. (2010). ReaxFF reactive force field development and applications for molecular dynamics simulations of ammonia borane dehydrogenation and combustion. J. Phys. Chem. A 114 (17), 5485–5492. 10.1021/jp100136c 20384351

[B22] Yang W. Zhu Z. Liao J. Liu Z. Gao F. Chen Y. (2024). ReaxFF-MD in the field of pyrolysis of insulating oil: a review. EDP Sci. 522, 01020. 10.1051/e3sconf/202452201020

[B23] Zhou T. T. Huang F. L. (2010). Effects of defects on thermal decomposition of HMX via ReaxFF molecular dynamics simulations. J. Phys. Chem. B 115 (2), 278–287. 10.1021/jp105805w 21142162

